# Association between the Presence of Class 1 Integrons, Virulence Genes, and Phylogenetic Groups of *Escherichia coli* Isolates from River Water

**DOI:** 10.1007/s00248-012-0101-3

**Published:** 2012-08-19

**Authors:** Ryszard Koczura, Joanna Mokracka, Agata Barczak, Natalia Krysiak, Adam Kaznowski

**Affiliations:** Department of Microbiology, Faculty of Biology, Adam Mickiewicz University in Poznań, ul. Umultowska 89, 61-614 Poznań, Poland

## Abstract

Ninety-six class 1 integron-positive and 96 integron-negative *Escherichia coli* isolates cultured from the water of the Warta River, Poland, were characterized for their phylogenetic group affiliation and for the presence of genes associated with virulence. Most strains belonged to phylogenetic group A, but phylogenetic group affiliation was not related with the presence of integrons. The occurrence of heat-stable toxin gene of enterotoxigenic *E. coli*, S fimbriae subunit gene *sfaS*, and siderophore receptor genes, *fyuA* and *iutA,* was associated with the presence of class 1 integrons. Moreover, virulence factor score (the total number of virulence-associated genes) was associated with the presence of integrons in groups. The results bring new insight into relations between the presence of integrons in *E. coli*, virulence traits, as well as phylogenetic group affiliation.

## Introduction

*Escherichia coli* is a part of intestinal flora of human and homoiothermal animals; however, pathogenic strains can cause gastrointestinal and extraintestinal infections. Strains that are responsible for gastrointestinal infections have been classified into several pathotypes, such as enteropathogenic *E. coli* (EPEC), enteroaggregative *E. coli* (EAEC), enterotoxigenic *E. coli* (ETEC), enteroinvasive *E. coli* (EIEC), diffusely adherent *E. coli* (DAEC), and Shiga toxin-producing *E. coli* (STEC), also referred to as enterohemorrhagic *E. coli* (EHEC). Major extraintestinal infections include mostly urinary tract infection, pyelonephritis, neonatal meningitis, cystitis, and bacteremia [1].

Pathogenic *E. coli* strains differ in their virulence factor (VFs) which confer pathogenic potential. Some recognized VFs of pathogenic *E. coli* include diverse adhesins (e.g., P fimbriae, S and F1C fimbriae), toxins (e.g., hemolysin and cytotoxic necrotizing factor), siderophores (e.g., aerobactin and yersiniabactin), polysaccharide coatings (e.g., group II and group III capsules and lipopolysaccharide), and invasins. These VFs alleviate colonization and invasion of the host, avoidance or disruption of host defense mechanism, and stimulation of inflammatory response [1].

Phylogenetically, strains of *E. coli* are divided into four groups: A, B1, B2, and D. They can be distinguished upon the presence of *chuA* and *yjaA* genes, and TSPE4.C2 noncoding region [2]. Some studies have reported an association of VF genes with phylogenetic group affiliation [1, 3, 4]. Commensal *E. coli* strains belong mostly to groups A and B1 9130. EHEC, ETEC, and EIEC strains are linked with groups A and B1 [5]. Strains isolated from extraintestinal infections, on the other hand, belong mostly to group B2 and, to a lesser extent, D [1, 5]. EPEC, EAEC, and DAEC are not associated with phylogenetic group affiliation [5]. The predominance of different phylogroups may also vary due to geographical location, the site of infection, and the level of antibiotic resistance [6, 7].

The presence of *E. coli* strains with virulence factors in water environments creates a potential risk of infections in humans due to the use of the water as a source of drinking or for recreation [8–10]. Another risk comes from the antibiotic resistance of the environmental *E. coli* strains. Multiresistant *E. coli* have been reported in river water worldwide [11–15]. An important role in the spread of antibiotic resistance is played by integrons—genetic platforms that are responsible for integration and rearrangements of resistance determinants called gene cassettes [16]. They consist of an integrase gene, a primary recombination site called *attI*, and a promoter P_C_ that directs transcription of the integrated genes. Three classes of integrons, distinguished upon the integrase gene sequences, are responsible for multidrug resistance, with class 1 being most ubiquitous among resistant bacteria and considered to play the main role in the emergence and widespread of resistance genes [16, 17]. Bacterial strains harboring integrons are usually multiresistant, i.e., resistant to antimicrobials of at least three different classes [12, 18, 19].

Some studies indicated that among clinical *E. coli* strains, the production of virulence factors is negatively associated with resistance to some antibiotics, including fluoroquinolones [20, 21]. On the other hand, such phenomenon is not observed among isolates of animal origin [20]. This suggests that ecological factors may determine relations between bacterial virulence and antibiotic resistance [20].

Chen et al. [22] have showed that *E coli* stains isolated from river water can carry both virulence genes and integrons. Little is known, however, about the relation between the occurrence of VF genes and the presence of integrons in environmental *E. coli* isolates. Hence, the aim of this study was to determine the presence of virulence-associated genes and the phylogenetic group affiliation of *E. coli* isolates cultured from river water, with regard to the presence of class 1 integrons.

## Materials and Methods

### Bacterial Strains and Class 1 Integrase Presence

A total of 192 isolates of *E. coli* cultured from the water of the Warta River, Poland, were used in this study. The Warta River runs in central–western Poland and has a length of 808 km. Water samples were collected three times in 2010, at eight stations localized along the river course. The samples were taken at the depth of 0.3 m and the distance of 1 m from the riverbank. The samples were then transported to a laboratory at 4 °C in a temperature-controlled box and analyzed within 8 h. *E. coli* strains were isolated on Brilliance E. coli/coliform Selective Agar (Oxoid) and identified with API 20E (bioMérieux). The isolates were then checked for the presence of integrase genes in the multiplex PCR assay according to Dillon et al. [23]. Ninety-six unique strains with class 1 integrase gene (*intI1*) were chosen from a pool of 279 isolates, and 96 integron-negative strains were chosen from a total of 3,417 isolates with the use of Research Randomizer 4.0 random number generator. The strains were selected randomly and proportionally to the number of strains per each sampling site. The strains used in the study were genetically unrelated: they had distinct DNA fingerprinting pattern (<90 % similarity, i.e., at least one band differing two isolates) determined in ERIC-PCR according to Versalovic et al. [24], followed by computer analysis of gels by using GelCompar II 3.5 software (Applied Maths) with Dice similarity coefficient and UPGMA clustering method.

### Analysis of Variable Region of Class 1 Integrons

The size of the variable regions of class 1 integrons was determined by PCR amplification with primers complementary to their 5′ and 3′ conserved regions [25]. The PCR was conducted as follows: initial denaturation 94 °C, 5 min, and 30 cycles of 94 °C at 1 min, 59 °C at 1 min, 72 °C at 5 min, and final elongation 72 °C at 8 min. After agarose gel electrophoresis with MassRuler DNA Ladder (Fermentas), the size of the PCR products was determined by using GelCompar II 3.5. The amplicons were then purified with ExoSAP-IT (Affymetrix) and sequenced in a 3100xl Genetic Analyzer (Applied Biosystems). Sequence data were assembled with DNA Baser (HeracleSoftware) and aligned with GenBank data by using nucleotide Basic Local Alignment Search Tool.

### Determination of Phylogenetic Groups

Affiliation of *E. coli* isolates to phylogenetic groups A, B1, B2, and D, and subgroups A_0_, A_1_, B1, B2_2_, B2_3_, D_1_, and D_2_, was done upon triplex PCR method according to Clermont et al. [2]. The PCR assay applied primers targeting genes chuA and yjaA, and a noncoding region Tspe4.C2. The reaction consisted of initial denaturation for 5 min at 94 °C and 30 cycles of 30 s at 94 °C, 30 s at 55 °C, and 30 s at 72 °C followed by a final extension step of 7 min at 72 °C. The four phylogenetic groups and seven subgroups were distinguished upon the following genotypes: A:A_0_, *chuA*-, *yjaA*-, Tspe4.C2- and A_1_, *chuA*-, *yjaA*+, Tspe4.C2-; B1, *chuA*-, *yjaA*-, Tspe4.C2+; B2:B2_2_, *chuA*+, *yjaA*+, Tspe4.C2- and B2_3_, *chuA*+, *yjaA*+, Tspe4.C2+; D:D_1_, *chuA*+, *yjaA*-, Tspe4.C2- and D_2,_ *chuA*+, *yjaA*-, Tspe4.C2+ [2, 26].

### Identification of Genes Associated with Virulence

Multiplex PCR assays according to Aranda et al. [27] were done to detect typical and atypical EPEC, EAEC, ETEC, EIEC, and STEC isolates. The targeted genes were *eae* and *bfpA* for EPEC; CVD432 for EAEC; heat-labile (LT) and heat-stable (ST) enterotoxin genes for ETEC; *ipaH* for EIEC; and *stx1* and *stx2* for STEC.

Virulence genes characteristic for extraintestinal *E. coli* were detected by PCR assays. The following genes were targeted using primers and PCR conditions recommended by Johnson and Stell [1]: *fyuA* (encoding yersiniabactin receptor), *iutA* (aerobactin receptor gene), *hlyA* (haemolysin A gene), *papA* (P fimbriae subunit gene), *sfaS* (S fimbriae subunit gene), *focG* (F1C fimbriae subunit gene), and *cnf1* (cytotoxic necrotizing factor gene). VF virulence factor gene score was determined as the total number of virulence-associated genes per strain.

### Statistical Analysis

Association between VF genes and the presence of class 1 integrase gene was determined with Pearson’s χ^2^ test or Fisher’s exact test, depending on cell frequencies, with *P* < 0.05 reflecting statistical significance. For comparisons on the prevalence of VF genes and integrase genes among phylogenetic groups, *P* value threshold was lowered to *P* < 0.008 according to Bonferroni’s correction for multiple comparisons. Association between VF scores and integron presence was determined with the Mann–Whitney *U* test. Association between VF scores and phylogenetic groups was established with Kruskal–Wallis test. Calculations were done with Statistica 10 (StatSoft).

## Results

The number of *E. coli* in the water samples taken from the Warta River during isolation of the study material ranged from 4.0 × 10^1^ to 3.6 × 10^4^ CFU/100 ml. A total of 192 *E. coli* isolates (96 with class 1 integrons and 96 without integrons) were analyzed for their phylogenetic group affiliation and the presence of virulence-associated genes.

The sizes of the variable regions of the integron-positive isolates ranged between 0.18 and 3.0 kbp. Sequence analysis of their content showed that the most frequent gene cassette array was *dfrA1*-*aadA1* (50.0 % of the strains) followed by *dfrA17*-*aadA5* (15.6 %), *aadA1* (13.5 %), *dfrA1* (5.2 %), *aacA4*-*aadA1* (3.1 %), *dfrA12*-*orfF*-*aadA* (3.1 %), and *aadB*-*aadA1*-*cmlA1* (1.0 %). Two strains (2.1 %) had variable regions of 0.18 kbp with no gene cassettes. For six strains (6.2 %), we did not manage to amplify the variable region. There was no correlation between the size of the integron variable regions and phylogenetic group affiliation and the presence of virulence-associated genes.

The most prevalent phylogenetic group in both *intI1*-positive and *intI1*-negative isolates was A (*P* < 0.01). However, there was no association between the presence of class 1 integrons and phylogenetic group affiliation. The frequencies of integron-positive and -negative isolates in four major phylogenetic groups were similar, with no statistically significant differences (Table 1).

**Table 1:** Phylogenetic group affiliation of integron-positive and -negative *E. coli* isolates from the Warta river

Phylogenetic group (*n*)	Class 1 integron presence		*P* value^c^
	*intI1*-positive, %^a^ (*n* ^b^)	*intI1*-negative, % (*n*)	
A (92)	45.7 (42)	54.3 (50)	0.248
A_0_ (23)	34.8 (8)	65.2 (15)	0.120
A_1_ (69)	49.3 (34)	50.7 (35)	0.880
B1 (37)	56.8 (21)	43.2 (16)	0.360
B2 (21)	57.1 (12)	42.9 (9)	0.488
B2_2_ (7)	85.7 (6)	14.3 (1)	0.118
B2_3_ (14)	42.9 (6)	57.1 (8)	0.579
D (42)	50.0 (21)	50.0 (21)	1.000
D_1_ (24)	33.3 (8)	66.7 (16)	0.081
D_2_ (18)	72.2 (13)	27.8 (5)	0.081

^**a**^Percentage of strains within a phylogenetic group/subgroup

^b^Number of strains

^c^ *P* value calculated in Pearson’s χ^2^ test or Fisher’s exact test (when cell frequency ≤5)

The presence of virulence-associated genes was determined in a series of PCR assays. Eighty-four (87.5 %) *intI1*-positive and 53 (55.2 %) *intI1*-negative isolates carried at least one virulence gene. The results shown in Table 2 revealed statistically significant differences between integron-positive and -negative *E. coli* isolates in frequencies of heat-stable toxin gene (ST) of enterotoxigenic *E. coli*, S fimbriae subunit gene *sfaS*, and siderophore receptor genes *fyuA* and *iutA*. All those genes were found more often in isolates harboring class 1 integrons, regardless of their gene cassette content.

**Table 2:** Association between prevalence of virulence-associated genes and the presence of integrons in *E. coli* strains isolated from the Warta river water

VF gene	Class 1 integron presence		*P*
	*intI1*-positive, % (*n*)	*intI1*-negative, % (*n*)	
*eae*	(0)	(0)	–
*bfpA*	(0)	(0)	–
CVD432	(0)	1.0 (1)	1.000
LT	2.1 (2)	(0)	0.497
ST	17.7 (17)	1.0 (1)	<0.001^a^
*stx1*	5.2 (5)	4.2 (4)	1.000
*stx2*	3.1 (3)	2.1 (2)	1.000
*ipaH*	7.3 (7)	13.6 (13)	0.156
*fyuA*	50 (48)	20.8 (20)	<0.001^a^
*iutA*	37.5 (36)	20.8 (20)	0.011^a^
*hlyA*	2.1 (2)	1.0 (1)	1.000
*papA*	7.3 (7)	2.1 (2)	0.169
*sfaS*	28.1 (27)	12.5 (12)	0.007^a^
*cnf1*	2.1 (2)	(0)	0.497
*focG*	2.1 (2)	3.8 (3)	1.000

^a^Significant *P* values (χ^2^ or Fisher’s exact test)

The distribution of virulence-associated genes among phylogenetic groups and subgroups was presented in Table 3. The *eae* and *bfpA* genes, absent in all isolates, and CVD432, present only in one isolate from B1 group, were excluded from the table. Only the presence of *fyuA* and *iutA* genes was associated with phylogenetic group affiliation (*P* < 0.005). The yersiniabactin receptor gene *fyuA* was most frequent in B2 group (compared to groups A, B1, and D), and the aerobactin receptor gene *iutA* was more frequent in B2 group than in A and B1. Analysis of distribution of VF genes within particular phylogenetic groups showed differences in group B2 in the case of the *iutA* gene which was more frequent in the subgroup B2_3_ (*P* = 0.017), and in group A in the case of the *fyuA* gene which was more frequent in the subgroup A_1_ (*P* = 0.030).

**Table 3:** Distribution of virulence factor genes among *E. coli* isolates of different phylogenetic groups and subgroups

Phylogenetic group/subgroup (*n*)	VF genes											
	LT, %^a^ (*n* ^b^)	ST, % (*n*)	*stx*_*1*_, % (*n*)	*stx*_*2*_, % (*n*)	*ipaH*, % (*n*)	*fyuA*, % (*n*)	*iutA*, % (*n*)	*hlyA*, % (*n*)	*papA*, % (*n*)	*sfaS*, % (*n*)	*focG*, % (*n*)	*cnf1*, % (*n*)
A (92)	1.1 (1)	9.8 (9)	5.4 (5)	3.3 (3)	12.0 (11)	26.1 (24)	19.6 (18)	1.1 (1)	4.3 (4)	20.7 (19)	1.1 (1)	2.2 %(2)
A_0_ (23)	0 (0)	8.7 (2)	4.3 (1)	0 (0)	17.4 (4)	8.7 (2)	8.7 (2)	4.3 (1)	0 (0)	17.4 (4)	0 (0)	0 (0)
A_1_ (69)	1.4 (1)	10.1 (7)	5.8 (4)	4.3 (3)	10.1 (7)	31.9 (22)	23.2 (16)	0 (0)	5.8 (4)	21.7 (15)	1.4 (1)	2.9 (2)
B1 (37)	0 (0)	16.2 (6)	5.4 (2)	0 (0)	8.1 (3)	35.1 (13)	18.9 (7)	2.7 (1)	2.7 (1)	18.9 (7)	0 (0)	0 (0)
B2 (21)	0 (0)	4.8 (1)	0 (0)	0 (0)	0 (0)	76.2 (16)	66.7 (14)	0 (0)	4.8 (1)	33.3 (7)	0 (0)	4.8 (1)
B2_2_ (7)	0 (0)	0 (0)	0 (0)	0 (0)	0 (0)	71.4 (5)	28.6 (2)	0 (0)	0 (0)	14.3 (1)	0 (0)	0 (0)
B2_3_ (14)	0 (0)	7.1 (1)	0 (0)	0 (0)	0 (0)	78.6 (11)	85.7 (12)	0 (0)	7.1 (1)	42.3 (6)	0 (0)	7.1 (1)
D (42)	2.4 (1)	4.8 (2)	4.8 (2)	4.8 (2)	14.3 (6)	35.7 (15)	40.5 (17)	2.4 (1)	7.1 (3)	14.3 (6)	2.4 (1)	4.8 (2)
D_1_ (24)	0 (0)	8.3 (2)	(0)	0 (0)	8.3 (2)	33.3 (8)	37.5 (9)	0 (0)	4.2 (1)	16.7 (4)	0 (0)	8.3 (2)
D_2_ (18)	5.6 (1)	(0)	11.1 (2)	11.1 (2)	22.2 (4)	38.9 (7)	44.4 (8)	5.6 (1)	11.1 (2)	11.1 (2)	5.6 (1)	0 (0)

^a^Percentage of strains within a phylogenetic group/subgroup

^b^Number of strains

Comparison of VF scores of integron-positive and -negative *E. coli* isolates showed that the VF score was associated with the presence of class 1 integrons (Table 4). Comparison of VF scores between *intI1*-positive and *intI1*-negative isolates within particular phylogenetic groups revealed statistically significant differences in groups A (both subgroups), B1, and D (only subgroup D_1_). On the other hand, VF score was not associated with the gene cassette content of the integrons (Kruskal–Wallis test, *P* = 0.697).

**Table 4:** VF scores of integron-positive and integron-negative *E. coli* in relation to phylogenetic group affiliation

Phylogenetic group/subgroup	VF score range		Median VF score		Average VF score		*P*
	*intI1*-positive	*intI1*-negative	*intI1*-positive	*intI1*-negative	*intI1*-positive	*intI1*-negative	
A	0–4	0–3	1	1	1.52	0.68	<0.001^a^
A_0_	1–3	0–1	1	0	1.38	0.33	0.004^a^
A_1_	0–4	0–3	1	1	1.56	0.83	0.015^a^
B1	0–3	0–2	1	0	1.57	0.50	0.003^a^
B2	0–3	1–4	1.5	2	1.75	2.11	0.522
B2_2_	0–3	1	1	1	1.17	1.00	1.000
B2_3_	1–3	1–4	2.5	2	2.33	2.25	0.846
D	0–5	0–3	1	1	1.90	0.86	0.008^a^
D_1_	0–4	0–2	2	0.5	2.12	0.69	0.011^a^
D_2_	1–5	0–3	1	1	1.77	1.40	0.767
Total	0–5	0–4	1	1	1.65	0.82	<0.001^a^

^a^Statistically significant differences between *intI1*-positive and *intI1*-negative isolates (Mann–Whitney *U* test)

Kruskal–Wallis analysis of variance showed that VF score was associated with phylogenetic group affiliation (*P* = 0.009) when integron-positive and -negative isolates were taken together. However, when VF scores were compared for *intI1*-positive and *intI1*-negative isolates separately, statistically significant differences were found only for isolates without integrons (*P* = 0.001). Multiple pairwise comparisons in that group showed that B2 isolates had the highest VF score compared to any other phylogenetic group (*P* = 0.004 vs. group A, *P* = 0.003 vs. group B1, and *P* = 0.046 vs. group D).

## Discussion

Resistance integrons are found frequently among clinical *E. coli* isolates [28, 29] and are also present in strains isolated from the environment [11, 13, 15, 22]. Class 1 integrons can be considered markers of antimicrobial multiresistance, even regardless of their gene cassette content, as they are often located in transferable plasmids and/or transposons carrying resistance genes [18, 19]. This also facilitates the spread of resistance not only within the same species but also to other genera [30]. In this study, we analyzed two sets of environmental *E. coli* isolates (with and without class 1 integrons) for their virulence potential and phylogenetic group affiliation. There was no difference in phylogenetic group distribution between isolates with class 1 integrons and those without the integrons. Most of the isolates, regardless of integron presence, belonged to phylogenetic group A, which includes commensal *E. coli* [1], but also EHEC, ETEC, and EIEC [5]. Group A strains have been also most prevalent among integron-bearing intestinal *E. coli* isolates from healthy subjects without antibiotic exposure [31]. On the other hand, Figueira et al. [32], who have analyzed *E. coli* isolated from wastewater treatment plant and surface water, found class 1 integrons significantly less prevalent in group A than in the others. Mokracka et al. [3] have reported that groups D and B1 isolates are more frequent in integron-positive *E. coli* (compared to integron-negative isolates) originated from a wastewater treatment plant.

In both *intI1*-positive and -negative *E. coli* isolates, majority of the strains harbored at least one virulence-associated gene, but they occurred more frequently in integron-positive isolates (*P* < 0.001). One third of integron-positive isolates and 22 % of integron-negative isolates had genes characteristic for diarrheagenic *E. coli* pathotypes: ETEC, STEC, EAEC, and EIEC. The presence of diarrheagenic *E. coli* in river water have been reported by Loukiadis at al. [33], who found STEC and EPEC isolates in French rivers in sampling points located upstream and downstream of the effluent discharges of slaughterhouses. STEC genes have been also found in recreational waters in Argentina [34], whereas EAEC and atypical EPEC were isolated from St. Clair River and Detroit River areas [8].

Four genes, the heat-stable toxin gene of enterotoxigenic *E. coli*, S fimbriae subunit gene *sfaS*, and siderophore receptor genes *fyuA* and *iutA*, were associated with the presence of class 1 integrons, whereas none of the genes investigated occurred more frequently in the *intI1*-negative isolates. This may suggest that at least in some cases, those virulence genes were localized within the same plasmids as the integrons. Moreover, VF scores, which reflect pathogenic potential of strains, were also higher in integron-bearing isolates. VF scores were also associated with phylogenetic group affiliation when both integron-positive and -negative isolates were pooled together. Interestingly, this association was due to significantly higher VF scores of subroup B2_3_ integron-negative isolates and was absent in strains with class 1 integrons. It is worth to note that B2_3_ strains originate from human and have not been found in animal feces [35].

The variable region of the integrons showed little diversity. Only five different gene cassette arrays were detected. Half of the strains had integrons with *dfrA1*-*aadA1* gene cassette array. These genes code for dihydrofolate reductase conferring resistance to trimethoprim and aminoglycoside adenylyltransferase conferring resistance to aminoglycosides, respectively. Two isolates had “empty” integron with no incorporated gene cassettes, and we did not manage to amplify the variable region for six strains, which suggests altered sequence or the lack of *sul1* gene in the 3′ conserved region [13].

Subgroups B2_2_ and B2_3_ showed heterogeneity when it comes to the presence of *iutA* (*P* = 0.017), whereas A_0_ and A_1_ differed in the frequency of the *fyuA* gene (*P* = 0.030). These genes code for aerobactin- and yersiniabactin-mediated iron acquisition systems, respectively, and are frequent among clinical *E. coli* isolates [1, 20, 36].

This paper reports a relationship between integrons, phylogenetic groups, and virulence traits in *E. coli* isolated from river water. We showed that, in *E. coli* isolates originated from river water, (a) the presence of class 1 integrons, regardless of their gene cassette content, was positively associated with the presence and the number of virulence genes; (b) the presence of class 1 integrons was not associated with phylogenetic group affiliation; and (c) the number of virulence genes was associated with phylogenetic group affiliation. Environmental water habitats, especially rivers and streams, are ideal vectors for the antibiotic resistance dissemination [37]. Virulence-associated genes are often located in plasmids or pathogenicity islands. Simultaneous presence of VF genes and class 1 integrons enhances possibility of spreading both virulence traits and antibiotic resistance via horizontal gene transfer. This may lead to ecological changes and predomination of virulent, antibiotic-resistant bacteria in the environment.
